# Neuropsychiatric Symptoms in Cerebral Amyloid Angiopathy: A Significant but Overlooked Association

**DOI:** 10.3390/brainsci15090959

**Published:** 2025-09-03

**Authors:** Polona Rus Prelog, Matija Zupan, Senta Frol

**Affiliations:** 1 Centre for Clinical Psychiatry, University Psychiatric Clinic Ljubljana, 1000 Ljubljana, Slovenia; polona.rus@psih-klinika.si; 2 Faculty of Medicine, University of Ljubljana, 1000 Ljubljana, Slovenia; matija.zupan@kclj.si; 3 Department of Vascular Neurology, University Medical Centre Ljubljana, 1000 Ljubljana, Slovenia

**Keywords:** cerebral amyloid angiopathy, neuropsychiatric symptoms, depression, anxiety, apathy, cognitive impairment, cerebral microbleeds, small vessel disease, intracerebral hemorrhage

## Abstract

Cerebral amyloid angiopathy (CAA) is an increasingly recognized cause of cognitive decline and lobar intracerebral hemorrhage in older adults. Recent research highlights that neuropsychiatric symptoms (NPSs)—including depression, anxiety, apathy, and irritability—are highly prevalent in CAA, often emerging prior to overt cognitive impairment or major vascular events. Compared to other cerebrovascular diseases, CAA presents a distinctive and multifaceted NPS profile, with symptoms closely linked to disease severity and neuroimaging biomarkers such as white matter hyperintensities and microbleeds. Critically, NPSs in CAA can complicate cognitive assessment and predict worse functional outcomes, yet remain underappreciated in clinical and research contexts. Management is complicated by pharmacologic risks—including heightened bleeding risk associated with SSRIs and novel anti-amyloid therapies—underscoring the need for individualized and multidisciplinary approaches. We highlight the urgent need for standardized NPS assessment, targeted research into mechanisms and treatment, and greater integration of neuropsychiatric evaluation into CAA care. We suggest that recognizing NPSs as core clinical features—not secondary complications—of CAA is essential to improving both patient outcomes and scientific understanding. Future studies should focus on longitudinal analyses, the development of tailored interventions, and robust comparative research to clarify the pathophysiology, clinical trajectory, and optimal management of NPSs in CAA.

In recent years, neuropsychiatric research has rapidly advanced our understanding of how age-related brain changes—including cerebrovascular dysfunction—influence neuropsychiatric symptoms (NPSs) in older adults. Cerebral amyloid angiopathy (CAA), characterized by progressive amyloid-β deposition in cortical and leptomeningeal vessels [1,2,3], exemplifies the intricate relationship between vascular pathology and neuropsychiatric manifestations. While CAA frequently co-occurs with Alzheimer’s disease (AD), neuropathological studies reveal that only around half of AD cases are accompanied by CAA, and substantial proportions of elderly have either CAA or AD alone [4]. Thus, CAA represents a distinct, age-related pathology that is not universal in AD, nor vice versa [5,6]. While CAA is established as a major cause of lobar intracerebral hemorrhage and cognitive decline in late life, accumulating evidence underscores a substantial and underrecognized burden of NPSs—depression, anxiety, apathy, and irritability—in this population [7,8].

Despite overt clinical implications, the prevalence, course, and impact of NPSs in CAA remain understudied. Recent informant-based studies utilizing the Neuropsychiatric Inventory Questionnaire (NPI-Q) reveal depressive symptoms or dysphoria in nearly half (up to 49%) of CAA patients, with anxiety present in 7–38% across cohorts [9,10]. Apathy and irritability each affect roughly one-third of patients, often co-occurring and potentially overlapping with mood symptoms, which further complicates clinical assessments [8,9,10]. Importantly, severity of NPSs appears tightly linked to CAA pathology burden [9]. Even presymptomatic CAA—particularly in men—may be accompanied by significant NPSs, with apathy predominating, suggesting these symptoms may precede cognitive decline [11]. However, the evidence for sex differences in NPSs in CAA is inconsistent across studies, so these findings require further validation. Furthermore, recent large-cohort data found no significant association between the severity of NPSs—including apathy—and CAA MRI burden scores, indicating that the link between NPSs and imaging markers of CAA is not yet fully established [9].

Compared to other cerebrovascular diseases, CAA may exhibit a relatively distinct NPS profile. Depression, apathy, and irritability are frequently reported in CAA—often affecting up to half of patients—and have been observed to emerge even before overt cognitive impairment or hemorrhagic events in some cohorts [9,10,12]. Some studies suggest that the severity and variety of NPSs in CAA are more pronounced and can be linked to imaging biomarkers such as white matter hyperintensities and microbleeds [8].

Extending these observations, recent prospective studies have quantified the relationship between CAA MRI burden and NPS severity. For example, each unit increase in total CAA MRI burden predicted a 1.14-fold increase in NPS count and a 0.57 unit greater NPS severity, with similar findings for white matter hyperintensity severity [11]. Longitudinal analyses further indicate that NPSs predict lower cognitive performance and may be linked to disease progression; however, the dynamic relationship between imaging markers, NPS, and clinical outcomes in CAA requires further study [9,11,13,14].

Hallucinations and psychosis remain rare in isolated CAA but may occur with comorbid Alzheimer’s disease [15]. Furthermore, NPS in CAA can also precede or occur independently from major stroke or dementia, possibly distinguishing CAA from other cerebrovascular conditions where NPS typically follow cognitive decline or acute vascular events. However, while these clinical features suggest that NPSs in CAA are more severe and multifaceted than in other small vessel diseases, direct comparative data remain scarce and overlapping symptom patterns have been documented in related conditions [9,11,16,17,18]. These potential differences highlight the need for further comparative research and for specific screening and management approaches tailored to CAA.

Clinical impact of CAA has important pathophysiological underpinnings; NPSs in CAA extend well beyond comorbidity: depressive and anxiety syndromes independently impair attention, memory, and executive function [19]. Notably, anxiety in amyloid-positive individuals nearly doubles the risk of mild cognitive impairment and confers strong prognostic import [17]. The severity of depressive symptoms is linked to amyloid load on neuroimaging and to chronic stress, which may exacerbate CAA progression [10]. These bi-directional relationships reinforce that NPS are core to CAA rather than mere correlates.

In neurodegenerative disorders broadly, NPSs predict functional impairment, faster decline, and increased mortality risk [9]. Systematic identification and management of NPSs in CAA is thus not only clinically relevant, but may inform prognosis and intervention.

However, accurate detection of NPSs represents a challenge. Grading of NPSs in CAA is hampered by heterogeneity in assessment tools and lack of standardization. Instruments including the Center for Epidemiologic Studies Depression Scale (CES-D), the Hospital Anxiety and Depression Scale (HADS), the Geriatric Depression Scale (GDS), and the NPI-Q are variably used, complicating cross-study comparison [12]. Consensus on validated, standardized instruments—including clear clinical cut-offs—is needed to enable robust epidemiological work and development of guidelines.

Therefore, we urge that neuropsychiatric symptom assessment be fully integrated into routine clinical management and research protocols in CAA, with harmonized use of well-validated tools such as the HADS, GDS, and NPI-Q, given their established reliability in cerebrovascular populations to ensure comparability and rigor. It should however be noted that the NPI-Q, in particular, relies on informant reports and may not capture the patient’s subjective experience. Similarly, self-report scales like the HADS and GDS may be influenced by cognitive impairment or lack of insight.

To facilitate the integration of standardized NPS assessment tools within diverse clinical environments, protocols such as the HADS, GDS, and NPI-Q should be incorporated into routine workflows in memory clinics, stroke services, and neurology practices. Effective implementation would benefit from targeted clinician training, use of validated translated and culturally adapted versions, and streamlined processes that limit added burden to both staff and patients. Addressing barriers—including limited time, varying staff familiarity with these instruments, and inconsistent access to informants—is essential to optimize uptake and ensure comprehensive, high-quality assessment of NPSs.

Treatments warrant further considerations; the effective management of depression and anxiety in CAA must balance efficacy with safety due to vascular fragility and increased hemorrhagic risk. While antidepressants (SSRIs) are widely used, emerging evidence associates SSRI use with increased risk of cerebral microbleeds in CAA [20,21]. Particular caution is warranted in clinical decision-making due to the heightened risk of bleeding in CAA, which may be further exacerbated in the presence of Alzheimer’s disease comorbidity [20]. With the advent of new Alzheimer’s disease therapies—such as anti-amyloid monoclonal antibodies, which themselves may carry a risk of amyloid-related imaging abnormalities (ARIAs) and bleeding—careful assessment of polypharmacy and hemorrhagic risk in this population is now even more critical [21,22,23]. It is important to note that CAA is characterized predominantly by vascular deposition of β-amyloid 1-40, while parenchymal amyloid plaques in Alzheimer’s disease are mainly composed of β-amyloid 1-42. Thus, anti-amyloid antibody therapies could, in theory, be tailored to selectively target parenchymal Aβ1-42 while sparing vascular Aβ1-40, which may influence both efficacy and safety of these treatments [24].

Patients with CAA and prior intracerebral hemorrhage may also demonstrate lower responsiveness to antidepressants, highlighting risk of treatment resistance and need for alternative strategies [20]. Non-pharmacological modalities—cognitive behavioral therapy, psychosocial support, and psychological counseling—are particularly valuable and should be prioritized as adjunctive or even first-line options [9]. Although no controlled studies have evaluated the effectiveness of cognitive-behavioral therapy or other structured psychotherapies specifically in patients with CAA, non-pharmacological interventions are recommended based on safety and positive data from broader cerebrovascular and dementia populations [11]. We advocate for multidisciplinary treatment approaches that integrate careful pharmacological management with tailored psychosocial and behavioral interventions (including psychological support, cognitive rehabilitation, and behavioral interventions), especially given the delicate risk–benefit balance in CAA.

For severe or treatment-refractory cases, among the emerging and advanced treatments, neuromodulatory interventions show promise. Electroconvulsive therapy (ECT), performed with careful blood pressure control, has proven effective and safe for severe depression in CAA without increased bleeding risk [25]. Similarly, repetitive transcranial magnetic stimulation (rTMS) provides symptom relief in CAA-associated depression and has been tolerated without worsening microbleeds in single-case reports [26]. Larger studies are required to confirm safety and generalizability. Future research should prioritize longitudinal, multicenter approaches—including randomized controlled trials—focused on both pharmacological and non-pharmacological interventions for NPS in CAA.

In conclusion, depression, anxiety, and related NPSs are highly prevalent, frequently overlooked, and clinically significant in CAA [9,10,11]. Their presence confounds cognitive assessment, predicts worse functional outcomes [27], and signals urgent need for comprehensive, standardized screening, diagnosis, and management. By recognizing NPSs as core features of CAA, alongside cognitive and hemorrhagic manifestations, we can advance both patient care and research in this increasingly relevant disorder. Greater harmonization, innovation in assessment, and focused research on the pathophysiology and treatment of NPS in CAA is warranted to drive future advances and ultimately improve outcomes. Longitudinal cohort studies of NPSs in CAA as prognostic markers and therapeutic targets, research into neurobiological mechanisms linking amyloid angiopathy, neuroinflammation, and NPS- and CAA-specific randomized controlled trials for both pharmacological and non-pharmacological treatments are urgently needed. Digital phenotyping, neuroimaging, and biomarker-based work may enable earlier detection and better, personalized care.

## Data Availability

Not applicable.

## Abbreviations

The following abbreviations are used in this manuscript:

CAA	Cerebral amyloid angiopathy
NPS	Neuropsychiatric symptoms
HADS	Hospital Anxiety and Depression Scale
GDS	Geriatric Depression Scale
NPI-Q	Neuropsychiatric Inventory Questionnaire
CES-D	Center for Epidemiologic Studies Depression Scale
